# Right ventricle anatomy can predict new onset ventricular tachycardia in patients with repaired tetralogy of Fallot

**DOI:** 10.1186/1532-429X-15-S1-O100

**Published:** 2013-01-30

**Authors:** Beatrice Bonello, Aleksander Kempny, Anselm Uebing, Wei Li, Philip J Kilner, Gerhard Diller, Dudley Pennell, Daryl Shore, Sabine Ernst, Michael Gatzoulis, Sonya V Babu-Narayan

**Affiliations:** 1 CHU Timone Marseille, Marseille, France; 2 CMR Department, Royal Brompton Hospital, London, UK; 3 Congenital Heart Disease, Royal Brompton Hospital, London, UK

## Background

Repaired tetralogy of Fallot (rtoF) patients are at risk ventricular tachyarrhythmia and sudden cardiac death. Risk stratification for arrhythmia remains difficult.

We aimed to investigate whether cardiac anatomy and function assessed by cardiac magnetic resonance imaging (CMR) predict arrhythmia.

## Methods

One-hundred-and-fifty-four adults with rtoF, median age 30.8 (21.9-40.2) years, were studied with a standardised protocol including cardiovascular magnetic resonance (CMR) and prospectively followed-up over median 5.6 (4.6-7.0) years for the pre-specified endpoints of new-onset ventricular tachyarrhythmia (sustained ventricular tachycardia/ventricular fibrillation).

## Results

Nine patients had ventricular tachyarrhythmia (6%) during follow-up. Patients who developed ventricular tachyarrhythmia were older (42.5 [34.9-50.2] vs. 29 [21-40] years; p=0.01), had a later repair (12.8 [6.2-13.9] vs. 4.4 [2-8] years; p=0.02), larger akinetic right ventricular outflow track (RVOT) region (Figure ) (length 55 [34-60] vs. 30 [20-40] mm; p=0.002) and a lower RV ejection fraction (42 [40-52] vs. 53 [51-55] %; p=0.01), compared to the other patients. On univariate Cox analysis, RVOT akinetic region length and RV ejection fraction were predictive of ventricular tachyarrhythmia. On stepwise Cox regression analysis, the RVOT akinetic region length was the only remaining predictor (Hazard ratio 1.05, 95% Confidence Interval 1.01-1.08 per mm; p=0.004). The survival ROC curve analysis indicated a cut-off value of 30mm as a predictor of VA during 6 year follow-up with an AUC of 0.77, sensitivity of 83% and specificity of 61%. RVOT akinetic area length >30mm predicted reduced VA-free survival (Logrank p=0.002).

**Figure 1:**
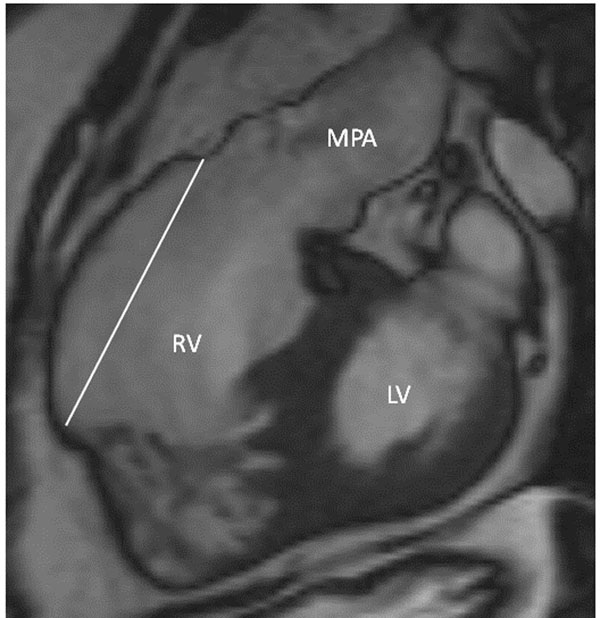
Cine steady state free precession right ventricular outflow track view showing a large akinetic area (50 mm).

## Conclusions

Conclusions: RVOT akinetic region length predicts ventricular arrhythmia in late follow-up of rtoF. This is simple, feasible measurements for inclusion in serial surveillance and risk stratification of rtoF patients.

## Funding

British Heart Foundation Fellowship (SVB-N).

French Federation of Cardiology (BB).

Unrestricted Actelion educational grant (GD).

The study was supported by the NIHR Cardiovascular Biomedical Research Unit of Royal Brompton and Harefield NHS Foundation Trust and Imperial College London.

